# Delineating the Immunotherapeutic Potential of Vitamin E and Its Analogues in Cancer: A Comprehensive Narrative Review

**DOI:** 10.1155/2024/5512422

**Published:** 2024-10-03

**Authors:** Nevvin Raaj Morgan, Premdass Ramdas, Saatheeyavaane Bhuvanendran, Ammu Kutty Radhakrishnan

**Affiliations:** Food as Medicine Research Strength Jeffrey Cheah School of Medicine and Health Sciences Monash University Malaysia, Bandar Sunway 47500, Selangor, Malaysia

**Keywords:** cancer immunity, immune modulation, tocopherol, tocotrienol, vitamin E

## Abstract

Cancer is a disease resulting from uncontrolled cell division, which significantly contributes to human mortality rates. An alternative approach to cancer treatment, such as cancer immunotherapy, is needed as the existing chemotherapy and radiotherapy approaches target the cancer cells and healthy dividing cells. Vitamin E is a plant-derived lipid-soluble antioxidant with numerous health-promoting benefits, including anticancer and immunomodulatory properties. Vitamin E comprises eight natural isoforms: tocopherols (*α*, *β*, *δ*, and *γ*) and tocotrienols (*α*, *β*, *δ*, and *γ*). While initial research focused on the anticancer properties of *α*-tocopherol, there is growing interest in other natural forms and modified synthetic analogues of vitamin E due to their unique properties and enhanced anticancer effects. Hence, this review is aimed at outlining the effect of vitamin E and its analogues at various steps of the cancer-immunity cycle that can be used to stimulate anticancer immune responses.

## 1. Introduction

Carcinogenesis, also known as oncogenesis, is the process whereby normal cells in the body undergo transformation into cancerous cells that are characterised by their ability to proliferate uncontrollably and metastasise, leading to various pathological conditions [1]. Cancer is a genetic disease because the uncontrolled division of cells is caused by changes in the genes responsible for regulating cell growth. According to the current statistics, cancer ranks as one of the three leading causes of death globally. Overall, lung cancer, female breast cancer (BC), and colorectal cancer show the highest incidence in both genders, while lung cancer and female BC are the leading causes of cancer-related mortality in males and females, respectively. Moreover, it is estimated that there will be an increase of 77% in cancer incidence by the year 2050 compared to 2022 [2]. Hence, a comprehensive understanding of the cancer pathogenesis and continued research on effective treatment options are vital to overcome this global burden. Surgical removal of tumours followed by radiotherapy and/or chemotherapy is the most commonly used cancer therapy [3]. However, radiotherapy and chemotherapy attack healthy cells, especially highly proliferative cells due to their inability to distinguish cancer and noncancer cells, resulting in unwanted toxicity [4]. Other than the lack of specificity and harmful side effects, another challenge in chemotherapy is drug resistance that is caused by reduced drug uptake and increased efflux of drugs by the cancer cells, calling for alternative treatments with better efficiency [5]. One of the most significant alternative treatment modalities in cancer treatment is immunotherapy, which involves inducing the immune system to generate anticancer immune responses.

**Figure 1:**
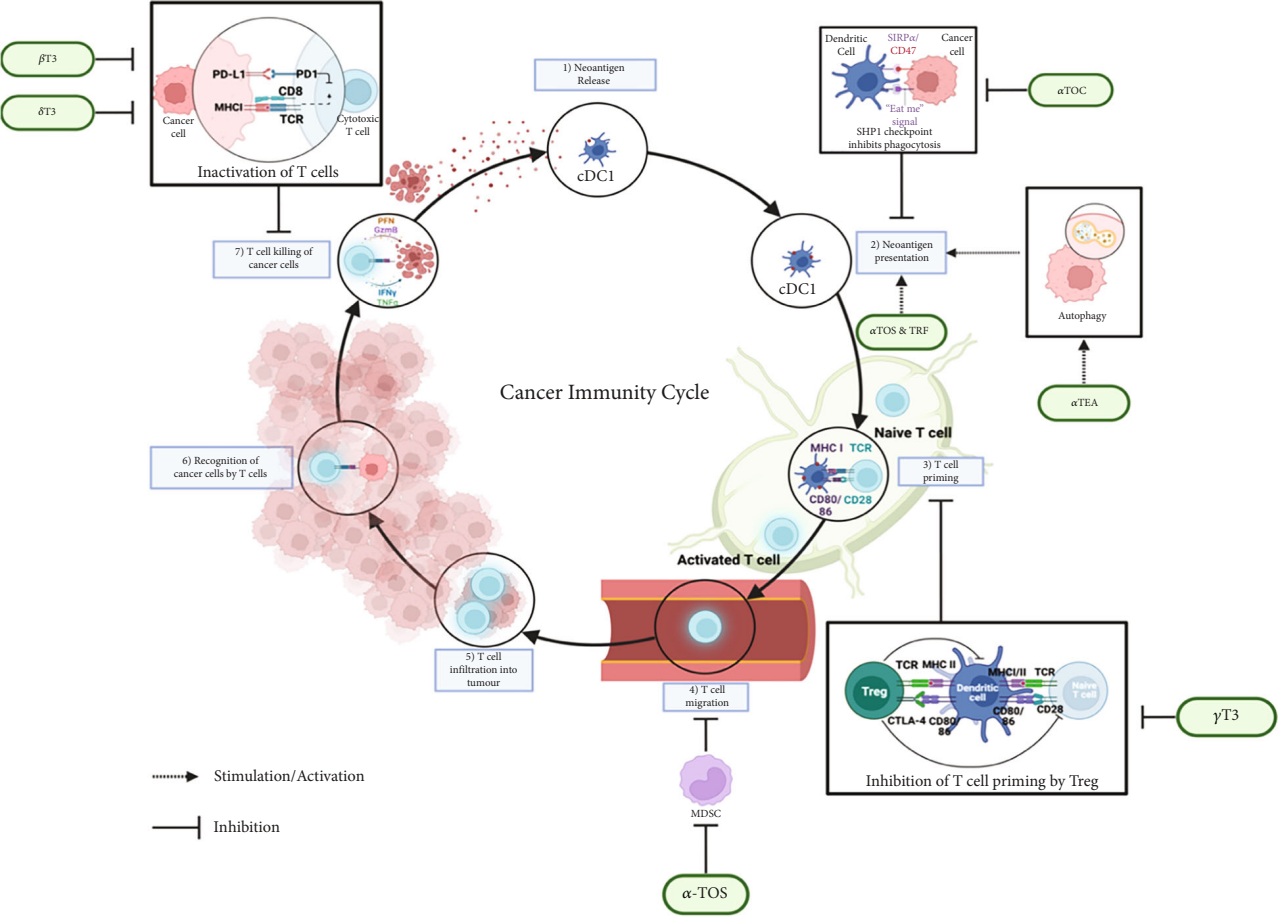
The various steps involved in the cancer-immunity cycle are shown in blue boxes. Step 1: release of cancer antigens; Step 2: neoantigen presentation by APC; Step 3: T-cell priming; Step 4: T-cell migration; Step 5: T-cell infiltrate into cancer; Step 6: T-cells recognise cancer cells; and Step 7: killing of cancer. The cancer immunity cycle can be modulated by different forms of vitamin E either directly or by inhibiting the immune evasion pathways, which augment anticancer immune response by inhibiting SHP1 checkpoint, directly enhancing neoantigen presentation, inhibiting Treg cells and MDSCs, and downregulating the expression of PD-L1. The steps modulated by the different forms of vitamin E are shown in green boxes. This figure was created using Biorender (https://biorender.com). *α*-TEA, *α*-tocopheryloxyacetic acid; *α*Toc, *α*-tocopherol; *α*TOS, *α*-tocopheryl succinate; *β*T3, beta-tocotrienol; CD, cluster of differentiation; cDC, classical dendritic cells; CTLA4, cytotoxic T-lymphocyte associated protein 4; DC, dendritic cell; *δ*T3, delta-tocotrienol; *γ*T3, gamma-tocotrienol; MDSC, myeloid-derived suppressor cell; MHC I, major histocompatibility complex class I; MHC II, major histocompatibility complex class II; PD-1, programmed cell death protein-1; PD-L1, programmed cell death protein ligand 1; SHP1, Src homology region 2 domain-containing phosphatase 1; SIRP*α*, signal regulatory protein-alpha; T3, tocotrienol; TCR, T-cell receptor; Treg, T-regulatory; TRF, tocotrienol-rich fraction.

Common types of immunotherapy include passive transfer of monoclonal antibodies, adoptive T-cell transfer (ACT) therapy, and immune checkpoint therapy [6]. The basis of immunotherapy for cancer is to strengthen the immune system which aids the immune cells to kill cancers and ensures the continuity of the cancer immunity cycle [4]. The concept of the cancer immunity cycle, first proposed by Chen and Mellman in 2013, represents a sequential event that leads to the effective killing of cancer cells by the host immune system in a self-propagating manner (Figure 1) [7]. The cancer immunity cycle involves seven sequential steps, starting with the release of neoantigen and culminating in the killing of cancer cells by T-cells. The tumour neoantigens or neoepitopes are proteins produced as a result of mutations within cancer cells and can manifest as foreign entities to the host immune system [8]. Therefore, the first step in the cancer immunity cycle is the release of cancer antigens from dying cancer cells. The dying cancer cells will be phagocytosed and processed for presentation by dendritic cells (DCs) or other professional antigen-presenting cells (APCs) (Step 2) (Figure 1). The DCs express transmembrane glycoproteins known as major histocompatibility complex (MHC) proteins on the cell surface. There are two classes of MHC that play a vital role in presenting peptides to T-cells, that is, MHC Class I (MHC I) and MHC Class II (MHC II). In general, MHC I proteins are found on the surface of most nucleated cells, while the expression of MHC II proteins is restricted to APC. The MHC I and MHC II present peptides from antigens to CD8^+^ and CD4^+^ T-cells, respectively.

DCs can activate tumour-specific T-cells that reside in the lymph nodes or other secondary lymphoid organs or tissues (Step 3) (Figure 1). The classical DC (cDC) that can activate CD4^+^ and CD8^+^ T-cells are known as Type 1 (cDC1) and Type 2 (cDC2) cells. The cDC1 plays a more important role in cancer immunity as these cells can directly activate CD8^+^ T-cells [9]. Presentation of antigens to CD8^+^ by cDC1 is known as cross-presentation or cross-priming, which is the activation of CD8^+^ T-cells by DCs that presents peptides from an extracellular protein source as opposed to the conventional intracellular peptides presented on the MHC Class I proteins [10].

Activation of naive T-cells requires three signals, which are (1) antigen-specific signal following the interaction of T-cell receptors (TCR) and antigen-loaded MHC molecules, which is followed by (2) signal generated through interactions between costimulatory molecules on the T-cells with their respective ligands on the APC. The third signal is the production of proinflammatory cytokines such as interferon-gamma (IFN-*γ*) and interleukin-12 (IL-12) [11]. Upon activation, the effector T-cells change their homing pattern (Step 4) by upregulating homing receptors that bind to ligands found in the tumour site, allowing them to infiltrate the tumour cells (Step 5) (Figure 1) [12]. Once the effector CD8^+^ T-cells, also known as the cytotoxic T-lymphocytes (CTLs), recognise tumour cells as foreign (Step 6), the tumour-specific killing process will be activated. Activated CTLs will release cytoplasmic granules that contain perforin and granzymes into the cancer cells, resulting in the death of the cancer cells (Step 7) [7] (Figure 1). Killing of the cancer cells releases more cancer antigens, and the immunity cycle will repeat.

**Figure 2:**
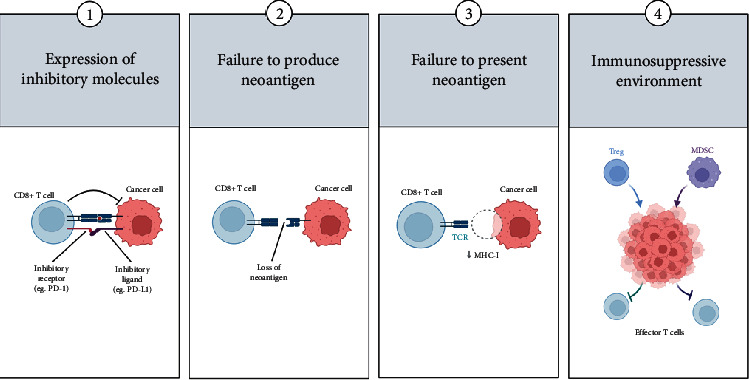
Some of the immune evasion mechanisms used by cancers. This figure was created using Biorender (https://biorender.com).

**Figure 3:**
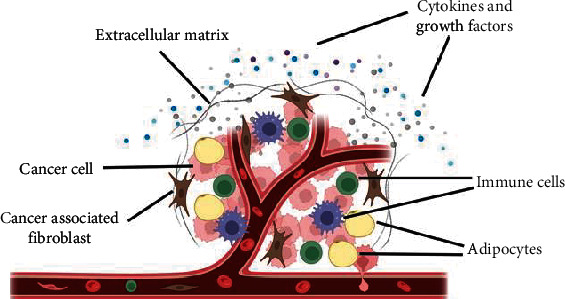
The key components that are usually found in the tumour microenvironment. This figure was created using Biorender (https://biorender.com).

However, in many cancer patients, the cancer-immunity cycle does not function optimally due to dysregulation of antigen presentation, deactivation of effector T-cells, and the presence of some immune-suppressive factors that are released in the tumour microenvironment (TME), which induce an influx of immunosuppressor cells that favours evasion of tumour immune surveillance (Figure 2), which is one of the 14 hallmarks of cancer [13, 14]. In humans, the TME is a heterogenous environment made up of several cellular components (e.g., cancer cells, immune cells, adipocytes, endothelial cells, and cancer-associated fibroblasts) and noncellular components (e.g., extracellular matrix, cytokines, chemokines, growth factors, apoptotic bodies, and exomes) (Figure 3) [15, 16]. Furthermore, it has been suggested that the TME is not just a “silent bystander” but rather an “active promoter of cancer progression” [17]. Hence, the goal of immunotherapy is to negatively modulate the negative regulators and support the stimulators of the cancer-immunity cycle to induce anticancer immune responses, resulting in an optimally functioning cancer-immunity cycle.

Studies on the biological activity of natural products and their potential use as an alternative treatment have gained importance in recent days as these bioactive compounds have little or no adverse effects [18]. Many of these natural bioactive products are also known for their immunomodulatory effects and potential benefits to enhance immune responses, as demonstrated by cell-based and animal model studies on their anticancer effects. Vitamin E is an example of a plant-derived bioactive compound with immunomodulatory and anticancer properties.

**Figure 4:**
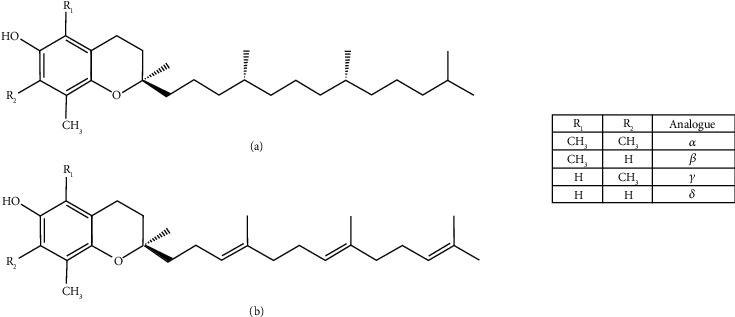
The chemical structures of (a) tocopherol and (b) tocotrienol and their analogues.

Vitamin E is a lipid-soluble antioxidant vitamin that exists naturally in two main forms, namely, tocopherols (Tocs) and tocotrienols (T3). Both forms of vitamin exist in nature in four analogues alpha (*α*), beta (*β*), delta (*δ*), or gamma (*γ*) [19]. The chemical structure of Tocs and T3s are similar, that is, both comprise a chromanol ring with a C2 side chain. The main difference between Tocs and T3s is that the side chain of Tocs is saturated, while the side chain of T3s contains three transdouble bonds in its phytyl tail (Figure 4). So, the Tocs are the “saturated” form of vitamin E, while the T3s are the unsaturated form of vitamin E. It is suggested that the presence of these unsaturated bonds in the phytyl tail of T3s enhances its ability to permeate organs compared to Tocs [20].

The major dietary sources of vitamin E are vegetable oils such as palm oil, sunflower oil, wheat germ oil, rice bran oil, vegetables (e.g., spinach), nuts, and fruits [21]. The Tocs and T3s are absorbed like how the human body absorbs lipids and are distributed lymphatically to peripheral tissues. However, it is reported that *α*-tocopherol (*α*Toc) undergoes a lower level of degradation compared to T3 as it binds to a carrier known as the *α*-tocopherol transfer protein (*α*TTP) in the plasma, thereby resulting in higher bioavailability [22]. In contrast, T3s are reported to have lower bioavailability as they lack affinity to *α*TTP and undergo rapid degradation. However, even with a low plasma concentration, the T3 has very good bioactivity [23]. In addition to its antioxidant properties, vitamin E is also known for reducing the risks of cardiovascular diseases, exerting anti-inflammatory and anticancer effects, and promoting skin health [19, 24–26]. Notably, among all the vitamins, vitamin E has a broader impact on the immune system and immune responses due to its higher concentration in immune cells compared to other blood cells [27]. The relatively higher concentration of vitamin E coupled with its potent antioxidant property maintains the membrane integrity of immune cells by preventing lipid peroxidation which ultimately aids in the normal functioning of immune cells, namely, in their proliferation and activation [28]. In addition, the anticancer properties of vitamin E have been reported across various cancers, with immunomodulation identified as one of the key mechanisms [29]. This review summarises and discusses the role of vitamin E and its analogues in augmenting anticancer immune response by affecting different key steps of the cancer-immunity cycle.

## 2. Search Methodology

The search and retrieval of the articles were done on electronic databases, Ovid MEDLINE, PubMed, and Google Scholar. Following are the keywords used in the article search: “Vitamin E,” “Tocopherols,” “Tocotrienols,” “Cancer,” “Neoplasms,” and “Immune.” Papers up to January 2024 were manually screened and selected by the authors, and only English-language papers were chosen. The inclusion criteria of the article selection were (i) cell and animal models, (ii) treatment with vitamin E or its analogues, and (iii) resulting in an anticancer immune response, while the exclusion criterion was the anticancer effect of vitamin E irrelevant to immune response.

## 3. Mechanisms of Anticancer Immune Response by Vitamin E and Its Analogues

Several studies have reported on the ability of vitamin E to enhance anticancer immune responses (Table 1). The various ways that vitamin E may augment the cancer immunity cycle are summarised in Figure 1. In this section, some of the reported anticancer immune response mechanisms augmented by various forms of vitamin E will be discussed.

### 3.1. Vitamin E Aids T-Cell Killing of Cancer

The goal of the anticancer immune response is to kill cancer cells by tumour-specific CTLs. The CTLs kill cancer cells by releasing granzymes and/or perforins, into the cancer cells, which activates apoptosis and disrupts the cell membrane integrity of target cells (in this case, cancer cells) [42]. The killing of cancer cells by CTLs is a form of cell-mediated immune response that is regulated by a subset of CD4^+^ T-cells known as the T-helper-1 (Th1) cells. Activated Th1 cells secrete IFN-*γ*, which activates CD8^+^ T-cells, enabling these cells to kill cancer cells, as well as activate macrophage-mediated cancer killing alongside inhibiting immune suppressive cells [43]. Moreover, Th1 cells also regulate cDC1 cells via the CD40L/CD40 interactions to cross-prime CD8^+^ T-cells [44]. More recently, immunotherapeutic strategies have been used to target and kill cancer cells, such as the ACT, which involves the bulk transport of syngeneic tumour-specific T-cells expanded ex vivo into cancer patients to specifically target and kill the cancers [45].

Some studies have found that supplementation with vitamin E can increase the population of T-cells in vivo. For instance, supplementation of *α*-tocopheryloxyacetic acid (*α*-TEA), a derivative of *α*Toc, showed a significant reduction in the tumour size, increased activated CD4^+^ and CD8^+^ T-cell populations, reduced the levels of the immunosuppressive T-regulatory (Treg) population, higher levels of IFN-*γ* secretion, and lower levels of IL-4 in a syngeneic mouse model of BC, which supports Th1 or cell-mediated immune response [30] (Table 1). Interestingly, an elevated level of the chemokine, C-C motif ligand 5 (CCL5), a chemoattractant, was also observed in the TME, which suggests that the T-cells were attracted to the tumour site [30, 46]. In another study, daily supplementation of *γ*T3 in a syngeneic mouse model of BC showed reduced tumour size, an increase in the CD4^+^CD127^+^ T-cell population, and a decrease in the Treg population alongside upregulation of tumour suppressor genes such as the *mitogen inducible gene 6* (*MIG6*) and *Cadherin-13* [31]. Furthermore, supplementation of *γ*T3 in the same mouse model of BC induced epigenetic changes in several genes, such as homeobox A10 (HOXA10) (reduced methylation), interferon-regulatory factor-4 (IRF4) (hypermethylation), and retinoic acid receptor–related orphan receptor alpha (ROR*α*) (hypermethylation) in CD4^+^ T-cells isolated from these animals [32] (Table 1). The upregulation of HOXA10 due to reduced methylation can be correlated to increased maturation and development of T-cells in the *γ*T3 supplemented mice [47]. Similarly, it has been reported that the downregulation of IRF4 [48] and ROR*α* [49] can enhance immune response via reducing the exhaustion of CD8^+^ T-cells and regulation of Th17 cells, respectively.

Vitamin E has also been used as a carrier of drugs to improve drug delivery to cancer cells. For instance, the use of vitamin E nanoemulsion as a carrier for paclitaxel (PTX) induced secretion of IL12 from macrophages, which is a form of Th1 response [50]. These findings suggest a dual role of vitamin E as a delivery agent of PTX and anticancer immune response inducer in metastatic BC treatment.

### 3.2. Vitamin E Enhances Neoantigen Presentation

One of the strategies used in cancer immunotherapy to enhance neoantigen presentation is the DC vaccine approach, as DCs are major APCs that facilitate the cross-talk between innate and adaptive immune systems [51]. The DC vaccines are syngeneic DCs pulsed with tumour antigens ex vivo before these are administered into the bodies of cancer patients or experimental animals [52]. However, the effectiveness of DC vaccines was found to be low, mainly due to tumour-mediated immunosuppression, needing more research on appropriate adjuvants to enhance the efficacy [53]. Vitamin E has been reported to support the cancer-immunity cycle by enhancing the first step, that is, the presentation of neoantigens by DC. A study using an analogue of vitamin E, *α*-tocopheryl succinate (*α*TOS), which is an esterified form of *α*Toc, showed that supplementation of *α*TOS with nonmatured DC reduced the size of the tumour in 3LL lung cancer-bearing C57BL/6 mice and increased the levels of IFN-*γ*, which again suggest the involvement of the Th1-mediated immune response [33]. In 2010, Hafid et al. [34] reported that daily supplementation of tocotrienol-rich fraction (TRF) can be used as an adjuvant candidate for DC vaccines in a syngeneic mouse model of BC. TRF is the vitamin E fraction isolated from palm oil, which contains approximately 75% T3s and 25% *α*Toc [54].

Cell-based studies showed that exposure to TRF decreased the viability of 4T1 murine mammary cancer cells but increased the viability of splenocytes and DC and increased the levels of IFN-*γ* and IL-12 [51]. IFN-*γ* and IL-12 are cytokines that promote cell-mediated activities, which induce the killing of cancers by activated CTL and natural killer (NK) cells [55]. Mice pretreated with DC pulsed with tumour lysate (TL) (DC+TL) together with daily TRF supplementation (DC+TL+TRF) prior to tumour induction inhibited tumour and significantly increased the CD8^+^ T-cells and NK populations as well as increased levels of IFN-*γ* and IL-12 [34]. In 2013, the same group tested the anticancer and antimetastatic abilities of DC+TL injections and daily supplementation of TRF in the same syngeneic mouse model of BC. The results showed that there was a significant reduction in tumour size and the number of metastatic deposits in the lung and higher levels of IFN-*γ* and IL-12, as well as increased CTL activity in tumour-induced mice treated with the DC+TL+TRF regime [35]. In addition, TRF supplementation also caused a significant increase in the CD40^+^, CD80^+^, CD83^+^, and CD86^+^ expressing leucocytes [35]. The increase in the CD40, CD80, CD83, and CD86 expressing cells suggests the maturation of DCs that drive the Th1 response [56, 57].

Another way of enhancing neoantigen presentation is via autophagy, which is a cellular process that clears worn-out organelles and infected cells through the formation of autophagosomes that are targeted for lysosomal degradation. In cancer immunity, autophagy can provide neoantigens needed for the priming of T-cells [58]. Li et al. [36] observed increased conversion of the microtubule-associated protein 1A/1B-light chain 3 (LC3) I to LC3-II, that is, stimulation of autophagy, in 3LL lung cancer cells and 4T1 murine BC cells treated with *α*TEA. A similar increase in the LC3-I to LC3-II conversion was also observed in mice induced with 4T1 BC fed with *α*TEA (6 mg/day) compared to the untreated tumour-induced mice. The increase in LC3-II formed indicates the increase in the formation of autophagosomes as they are well correlated [59]. Moreover, an autophagosome-enriched fraction (*α*-TAGS) from *α*-TEA-treated 3LL and 4T1 resulted in the efficient cross-presentation of neoantigens to T-cells and resulted in a significant reduction in the metastasis to lungs as well as reduced size of the tumour, increasing the survival in tumour bearing mice [36]. Similarly, induction of autophagy was also observed in *γ*T3-treated BC cell lines (e.g., SA+, MCF-7, and MDA-MB-231), as indicated by the LC3-I to LC3-II conversion [60]. However, autophagy is also reported to impair the efficiency of immunotherapy in certain cancers, calling for more research on vitamin E-induced autophagy and its impact on immune responses in different types of cancer [58, 61].

More recently, *α*Toc was reported to target the Src homology region 2 domain-containing phosphatase 1 (SHP1) checkpoint, which is an intrinsic checkpoint of DCs [37, 62]. The SHP1 is a type of phosphatase that can suppress the phagocytic ability of DCs, which takes place via interactions between the signal regulatory protein-alpha (SIRP*α*), which is expressed on DCs and macrophages and CD47, a cell surface protein expressed by tumours that induce the recruitment of SHP1 [63]. It is reported that *α*Toc enters DCs through the scavenger receptor class B type 1 (SCARB1) receptor and suppresses the expression of SHP1 through the SCARB1 receptor, which increases infiltration of CD11^+^cDCs and CD8^+^ T-cells into the TME, and this was found to enhance polarisation of the DCs to cross-presenting type in an MT6 orthotopic mouse model [37] (Table 1). The data from this study also suggest that inhibition of SHP1 expression by *α*Toc permits T-cell cross-priming and promotes their activation via increased accumulation of Ras-related protein Rab-34 (RAB34) and reduced fusion of phagolysosomes. Cross-presentation or cross-priming of CD8^+^ T-cells is promoted via the reduction of phagolysosome fusion by RAB34-dependent lysosomal reorganisation [64].

### 3.3. Vitamin E Reduces Suppressive Immune Cell Infiltration

One of the factors that contribute to the immune evasion by cancer cells is the recruitment of immunosuppressive cells such as Treg cells and myeloid-derived suppressor cells (MDSCs) that can negatively regulate effector T-cells in the TME [65]. One of the promising strategies to improve current immunotherapy protocols is to reprogramme the TME by targeting these immunosuppressive cells, which increases the effectiveness of effector T-cell functions. The MDSCs are of neutrophil and monocyte origin that have been transformed pathologically with immunosuppressive potential and are divided into granulocytic and monocytic types, respectively [66]. The MDSCs facilitate tumour immune evasion by interfering with the recruitment of lymphocytes to the TME, producing reactive oxygen (ROS) and nitrogen (RNS) species, and via depletion of T-cell metabolites [67]. In a separate study, it was shown that supplementation of D-*α*-tocopherol succinate (2 mg/kg body weight) inhibited the immunosuppressive activities of MDSCs in lung cancer-bearing C57BL/6 mice, where the authors reported a reduction in the tumour size and infiltration by MDSCs in the TME and increased levels of CD8^+^ T-cells in the tumours from vitamin E-treated mice [38] (Table 1). These observations suggest that the amelioration of MDSCs-mediated T-cell suppression by vitamin E may be attributed to its antioxidant property that can reduce the concentration of nitric oxide (NO). Increased concentrations of NO are linked with reduced T-cell function by MDSCs due to the high-level expression of NO synthase [68].

The Treg cells are a subpopulation of CD4^+^ cells that primarily functions to maintain immune balance and immunological tolerance and is characterised by the expression of a transcription factor known as the Forkhead box P3 (FoxP3), cytotoxic T-lymphocyte-associated protein-4 (CTLA-4), and CD25 [69]. The TME contains high levels of chemokines such as CCL5, C-C motif chemokine 22 (CCL22), and C-X-C motif chemokine 12 (CXCL12), which can attract Treg cells to the TME. The Treg cells hinder immunosurveillance in cancers by suppressing APCs via CTLA-4 engagement, depleting interleukin-2 (IL-2) that is needed for the expansion effector T-cells and producing immunosuppressive cytokines such as transforming growth factor-beta (TGF-*β*), interleukin-10 (IL-10), and interleukin-35 (IL-35) [70].

Recently, Subramaniam et al. studied the effect of *γ*T3, a subtype of T3, on the infiltration of Treg cells in a syngeneic mouse model of BC. Supplementation of *γ*T3 caused a significant increase in the CD4^+^, CD8^+^, and NK cells population while abating the population of Treg cells with a marked increase in the IFN-*γ* levels and a decrease in TGF-*β* levels [38]. Immunohistochemistry study showed reduced infiltration of FoxP3^+^ Treg cells and increased numbers of CD4^+^ T-cells and cells expressing interleukin-12-beta-2 receptor (IL-12R*β*2^+^) and interleukin-24 (IL-24^+^) in the tumour from *γ*T3-treated animals. Furthermore, gene expression analysis of the CD4^+^ T-cells from *γ*T3-treated animals showed downregulation of genes related to Th differentiation and immunomodulation, namely, *Jak1, Maf, Stat1, Socs1, Tlr4, Tlr6*, and *IL18* [39].

### 3.4. Vitamin E Inhibits T-Cell Inhibitory Molecules

One of the ways that cancer cells evade the host immune system is via the immune checkpoints such as programmed cell death protein-1 (PD1)-programmed cell death ligand 1 (PD-L1) pathway. The interaction between overexpressed PD1 (also known as CD279) on tumour infiltrating lymphocytes (TILs) due to overstimulation and PD-L1 (also known as CD274 or B7-H1) expressed on tumour cells leads to inactivation of the TILs [71, 72]. Inactivation of the TILs affects the proliferation of T-cells, and its activity is due to the dephosphorylation of the TCR resulting from the PD-L1/PD-1 axis [73]. Recently, Sun et al. [40] investigated the effects of *β*T3 on the expression of PD-L1 in LLC lung cancer and DU145 prostate cancer cell lines. The results from this study showed that treatment with *β*T3 reduced the levels of PD-L1 mRNA, PD-L1/PD-1 interactions, and expression of the signal transducer and activator of transcription 3 (STAT3) and Janus kinase 2 (JAK2) phosphorylation, which are linked to the transcription of PD-L1 [74]. A similar finding was observed in the LLC xenograft C57BL/6 N mouse model, which showed that treatment with *β*T3 reduced tumour size, reduced PD-L1 expression, as well as inhibition of JAK2/STAT3 pathway, increased CD8^+^ T-cell population and elevated granzyme B levels [40] (Table 1). A recent study using liquid chromatography and double mass-spectrometry (LC-MS/MS) reported that the membrane-bound PD-L1 in MDA-MB-231 human BC cells exhibited high glycosylation, specifically polylactic glycans at N219 sequon [75]. Glycosylation is a type of post-translational where glycans (chains of carbohydrate or sugar) are enzymatically added to proteins [76]. It was shown that glycosylation is needed in the stabilisation of PD-L1, and the interaction with PD-1 leads to immune suppression [77, 78]. It has been shown that the N-glycosylation of PD-L1 leads to better anti-PD-L1/PD-1 treatments in human lung and basal-like BC [79]. In 2024, Sun et al. [41] investigated the effect of *δ*T3 on the glycosylation of PD-L1 (Table 1). The results show that exposure to *δ*T3 disrupted the glycosylation in PD-L1 in A375 melanoma cells and DU145 prostate cancer cells. The disruption of glycosylation *δ*T3 in these two cancer cell lines led to lower PD-L1 expression and exosomal secretion and reduction of PD-L1/PD-1 interaction as seen in the T-cell killing assay using coculture of Jurkat T-cells and *δ*T3 pretreated DU145 cells. Furthermore, it was found that oral administration of *δ*T3 (100 mg/kg/d) for 12 days on LLC xenografts-bearing mice disrupted the glycosylation of PD-L1, reduced the expression of membrane-bound PD-L1, increased the ratio of CD8^+^ T-cells to CD4^+^ T-cells and increased the levels of granzyme B [41]. In addition, the mechanistic study in the same paper revealed decreased levels of STT3 oligosaccharyltransferase complex catalytic subunit A (STTa) and B (STTb) mRNA levels and inhibition of their transcription factor, transcription factor 4 (TCF4), which are linked to the downregulation of PD-L1 via inhibition of STT3-dependent N-glycosylation [41, 80]. However, it was reported that *α*Toc upregulates the expression of PD-L1 and activates the PD-L1/PD-1 axis via JAK/STAT3 and ERK pathways, resulting in tumour growth in the LLC xenograft mice [81].

## 4. Conclusion and Future Directions

In conclusion, studies have highlighted the potential of vitamin E and its analogues as immunotherapeutic agents in cancer. They achieve this by increasing the population of effector T-cells and inhibiting immunosuppressive cells as well as T-cell inhibitory molecules. However, while vitamin E's antioxidant, antiproliferative, apoptosis-inducing, and antiangiogenic properties contribute to its anticancer effects, limited research has focused on its immune modulation mechanisms in cancers. More in-depth mechanistic studies, both in cell-based systems and animal models, are necessary before translation to human applications. Additionally, comparative analyses using different isomers and analogues of vitamin E are crucial to identifying the most effective candidates for cancer immunotherapy. Understanding how different vitamin E types affect various cancer subtypes is also essential, as responses may vary. Challenges in vitamin E immunotherapy for patients include formulation, dosage optimization, delivery methods, and long-term safety profiles. To bridge the bench-to-bedside gap, pharmacokinetic and toxicity studies are vital to improve vitamin E delivery, assess bioavailability and biodistribution, and evaluate organ-system effects. Exploring predictive biomarkers related to vitamin E immune modulation could enhance treatment outcomes in clinical studies. Furthermore, evaluating vitamin E's potential as an adjuvant in combination therapy with chemotherapy, radiotherapy, or existing immunotherapies at both preclinical and clinical levels is warranted.

## Figures and Tables

**Table 1:** Summary of anticancer immune response by vitamin E and its analogues.

**Vitamin E**	**Cell/animal model used**	**Type of cancer**	**Principle outcome**	**Cytokine/chemokine/Granzyme B changes**	**Ref.**
*α*TEA	4 T1 tumour-bearing BALB/c mice	Breast cancer	↑ Activated CD4^+^ and CD8^+^ T-cells	↑ IFN-*γ*, ↓ IL-4, and ↑ CCL5	[30]
*γ*T3	4 T1 tumour-bearing BALB/c mice	Breast cancer	↑ CD4/CD127^+^ T-cells population and ↓ Treg population	Not reported	[31]
*γ*T3	4 T1 tumour-bearing BALB/c mice	Breast cancer	↓ HOXA10 methylation, ↑ IRF4 methylation, and ↑ ROR*α* methylation in isolated CD4^+^ T-cells	Not reported	[32]
*α*-TOS	3LL carcinoma bearing C57BL/6	Lung cancer	Adjuvant in DC vaccine	Not reported	[33]
TRF	4 T1 tumour-bearing BALB/c mice	Breast cancer	Adjuvant in DC vaccine	↑ IFN-*γ*, ↑ IL-12	[34, 35]
*α*-TEA	3LL and 4 T1 cell lines	Lung cancer and breast cancer	Stimulates autophagy	Not reported	[36]
	4 T1 tumour-bearing BALB/c mice	Breast cancer			
*α*-Toc	EMT6 orthotopic mouse model	Breast cancer	Activates DC by blocking SHP1 checkpoint	Not reported	[37]
D-*α*-tocopherol succinate	TC-1 tumour bearing C57BL/6	Lung cancer	↓ Infiltration of MDSC	Not reported	[38]
*γ*T3	4 T1 tumour bearing BALB/c	Breast cancer	↓ Infiltration of Treg	↑ IFN-*γ*, ↓ TGF-*β*	[39]
*β*T3	LLC xenograft C57BL/6 N	Lung cancer	↓ PD-L1 expression	↑ Granzyme B	[40]
*δ*T3	LLC xenograft C57BL/6 N	Lung cancer	↓ PD-L1 expression	↑ Granzyme B	[41]

*Note:* ↑: increase; ↓: decrease.

Abbreviations: *α*-TEA: *α*-tocopheryloxyacetic acid; *α*Toc: *α*-tocopherol; *α*TOS: *α*-tocopheryl succinate; *β*T3: beta-tocotrienol; CCL5: chemokine (C-C motif) ligand 5; *δ*T3: delta-tocotrienol; DC: dendritic cell: *γ*T3: gamma-tocotrienol; HOXA10: homeobox A10; IFN-*γ*: interferon-gamma; IL-4: interleukin-4; IL-12: interleukin-12; IRF4: interferon-regulatory factor-4; MDSC: myeloid-derived suppressor cell; PD-1: programmed cell death protein-1; PD-L1: programmed cell death protein ligand 1; ROR*α*: retinoic acid receptor-related orphan receptor alpha; SHP1: Src homology region 2 domain-containing phosphatase 1; T3: tocotrienol; TGF-*β*: transforming growth factor-beta; Treg: T-regulatory cells; TRF: tocotrienol-rich fraction.

## Data Availability

Data sharing is not applicable to this article as no new data were created or analyzed in this study.

## Nomenclature

*α*-TEA: *α*-tocopheryloxyacetic acid
*α*Toc: *α*-tocopherol
*α*TOS: *α-*tocopheryl succinate
*α*TTP: *α*-tocopherol transfer protein
ACT: adoptive T-cell transfer
APC: antigen-presenting cell
BC: breast cancer
CCL5: chemokine (C-C motif) ligand 5
CD: cluster of differentiation
cDC 1: type 1 classical dendritic cell
cDC 2: type 2 classical dendritic cell
CTL: cytotoxic T-lymphocyte
CTLA-4: cytotoxic T-lymphocyte associated protein 4
DC: dendritic cell
ERK: extracellular signal-regulated kinases
HOXA10: homeobox A10
IFN-*γ*: Interferon gamma
IL-12: interleukin 12
IL-18: interleukin 18
IRF4: interferon-regulatory factor-4
JAK: Janus kinases
LC3: microtubule-associated protein 1A/1B-light chain 3
MDSC: myeloid-derived suppressor cell
MHC: major histocompatibility complex
NK cell: natural killer cell
PD-1: programmed cell death protein1
PD-L1: programmed cell death protein ligand 1
PTX: paclitaxel
RNS: reactive nitrogen species
ROR*α*: retinoic acid receptor-related orphan receptor alpha
ROS: reactive oxygen species
SHP1: Src homology region 2 domain-containing phosphatase 1
Socs1: suppressor of cytokine signalling 1
STAT: Signal transducer and activator of transcription
T3: tocotrienol
TCR: T-cell receptors
Th: T helper cells
TL: tumour lysate
Tlr: Toll-like receptor
TME: tumour microenvironment
Toc: tocopherol
T_regs_: regulatory T-cells
TRF: tocotrienol-rich fraction
